# Development of a Double-Antibody Sandwich ELISA for Rapid Detection of the MCP Antigen Concentration in Inactivated ISKNV Vaccines

**DOI:** 10.3390/vaccines9111264

**Published:** 2021-11-02

**Authors:** Hongru Liang, Lixi Zhang, Xiaozhe Fu, Qiang Lin, Lihui Liu, Yinjie Niu, Xia Luo, Zhibin Huang, Ningqiu Li

**Affiliations:** Pearl River Fisheries Research Institute, Chinese Academy of Fishery Sciences, Key Laboratory of Fishery Drug Development, Ministry of Agriculture and Rural Affairs, Guangdong Province Key Laboratory of Aquatic Animal Immune Technology, Guangzhou 510380, China; hrliang10@hotmail.com (H.L.); zhanglixi2021@163.com (L.Z.); fuxiaozhe-1998@163.com (X.F.); lin9902057@163.com (Q.L.); lhliu0614@163.com (L.L.); niuyinjie0530@163.com (Y.N.); lxwenhao@163.com (X.L.); hzb1393@163.com (Z.H.)

**Keywords:** ISKNV, double-antibody sandwich ELISA, monoclonal antibody, inactivated vaccine, antigen concentration

## Abstract

Infectious spleen and kidney necrosis virus (ISKNV) resulted in severe systemic diseases with high morbidity and mortality in Siniperca chuatsi. Vaccination is the primary method for effective prevention and control of these diseases. The development of inactivated ISKNV vaccines made some progress, but the technique of quality evaluation is scarce. Herein, a measurement of the MCP (major capsid protein) antigen concentration for the inactivated ISKNV vaccine was developed by double-antibody sandwich ELISA. Firstly, mouse monoclonal antibodies against ISKNV particles and MCP were generated. Then, a double-antibody sandwich ELISA was developed using the monoclonal antibody 1C8 1B9 as the capture antibody and Biotin-3B12 6B3 as the detection antibody. A standard curve was generated using the MCP concentration versus OD value with the linear range of concentration of 4.69~300 ng/mL. The assay sensitivity was 0.9 ng/mL. The antigen content of three batches of inactivated ISKNV vaccines was quantitatively detected using the double-antibody sandwich ELISA. The results showed that MCP antigen contents of inactivated ISKNV vaccines were positively correlated with the viral titers. The newly established double-antibody sandwich ELISA provided a useful tool for the detection of antigen quality for ISKNV inactivated vaccines.

## 1. Introduction

Infectious spleen and kidney necrosis virus (ISKNV) infections occurred in more than 50 species of fish, including freshwater and marine species [1]. Affected fish presents with depression, lethargy, pale body pigmentation and gill pallor. In China, ISKNV infection results in high mortality and economic loss in Chinese perch, *Siniperca chuatsi* [2]. To date, there is no effective antiviral treatment against infectious spleen and kidney necrosis virus infection. Vaccination is one of the most practical and effective measures to prevent outbreaks of ISKNV. The major capsid protein is the most abundant viral protein in all members of iridoviruses and accounts for about 40% of total virion proteins [3,4]. 

The concentration of the MCP antigen in the inactivated ISKNV vaccine is very important for the efficacy of the vaccine. Therefore, a quality evaluation must detect protective antigen concentration in inactivated vaccines. However, there is no effective test to measure the MCP antigen concentration for inactivated ISKNV.

The enzyme-linked immunosorbent assay (ELISA) was extensively used to measure antigen concentration in viral vaccines because of its high specificity and sensitivity [5]. In this study, mouse monoclonal antibodies against ISKNV were generated. Then, the coating antibody and the capture antibody were selected and optimized. Subsequently, an antibody sandwich ELISA was developed to detect the MCP antigen concentration in the inactivated ISKNV vaccine. The results showed that this assay was sensitive, convenient and accurate in the detection of antigen concentrations of inactivated ISKNV vaccines.

## 2. Materials and Methods

### 2.1. Cells, Animals and Reagents

The Chinese perch brain (CPB) cell line [6], SP2/0 cell line and ISKNV were stored in our laboratory. Six- to eight-week-old female specific pathogen-free BALB/c mice were purchased from Hubei Experimental Animal Research Center. CPB cells were cultured at 28 °C in L-15 medium (Invitrogen, Carlsbad, CA, USA) supplemented with 10% fetal calf serum (FBS; HyClone, Logan City, UT, USA).

### 2.2. Virus Proliferation and Purification

#### 2.2.1. Virus Production

CPB cells were infected with ISKNV at an MOI (multiplicity of infection) of 1 in L-15 medium containing 5% heat-inactivated fetal bovine serum (FBS) and were cultured at 28 °C. Upon observation of the cytopathic effects, the cell culture supernatants were collected, and freeze–thaw cycles were repeated three times. The viral titer was measured by Reed-Muench as described previously [7].

#### 2.2.2. Sucrose Density Gradient Centrifugation

Cell culture supernatants were collected and clarified by centrifugation at 5000× *g* for 30 min. The gradients were centrifuged for 1 h at 200,000× *g*. The viral particles were separated as a single band. The purified viruses were determined by electronic microscopy (Hitachi HT7800; Hitachi High-Tech Science Corporation, Tokio, Japan) observation and were electrophoresed on SDS-PAGE gels.

### 2.3. Preparation and Identification of Monoclonal Antibody (mAbs) against ISKNV

#### 2.3.1. Animal Immunization

The 6–8-week-old SPF female BALB/c mice were intraperitoneally injected with purified ISKNV virus emulsified with Freund’s complete adjuvant. Subsequently, the mice were immunized with 50 μg/mice purified virus emulsified with Freund’s incomplete adjuvant three times at 2-week intervals. Three days after the fourth immunization, antiserum was collected from the immunized mice and titers were monitored by in-direct-ELISA. Two weeks after the fourth immunization, the mice were injected intraperitoneally with 50 μg/mice antigen in sterile PBS, and cell fusion was conducted on three days post-vaccination (Table 1).

#### 2.3.2. Cell Fusion

Three days after the last immunization, the spleen cells of BALB/c mice and the murine myeloma cells (SP2/0) were mixed together at a ratio of 10:1. Then, the cells were incubated in 50% PEG1450 for 1 min, which was stopped by the addition of DMEM. After centrifugation, the pelleted cells were gently suspended in hypoxanthine–aminopterin–thymidine (HAT) medium containing 20% FBS. The fusion cells were added to the cell culture plates, which were paved with feeding cells, and were incubated in 5% CO_2_ at 37 °C.

#### 2.3.3. Hybridoma Screening

Hybridoma clones were selected in the HAT medium, and the resulting supernatants were screened using an indirect ELISA. The indirect ELISA did as follows: the 96-well microtiter plates were coated with purified viruses (4 μg/mL) overnight at 4 °C. After washing three times with PBST, the plates were blocked for 1 h at 37 °C. The cell supernatants were added to each well and incubated for 1 h at 37 °C. Then washing three times with PBST, the plates were incubated with horseradish peroxidase (HRP)-conjugated goat anti-mouse IgG (1:10,000, Jackson, America) for 1 h at 37 °C. After washing, the plate was inoculated with a chromogen reagent, and the absorbance was measured at 450 nm. The positive hybridoma cells were screened and cloned using the limited dilution method. Then, the hybridoma cells (2 × 10^6^) were intraperitoneally injected into 7-week-old BALB/c mice. Seven days later, the ascites were collected and purified.

#### 2.3.4. Characterization of the mAbs

There were different subtypes of IgG. The subclasses of the mAbs were determined using the mouse monoclonal antibody iso-typing kit (Sigma Aldrich, Burlington, MA, USA). As described in the kit, when the OD value was more than 1.5, it was regarded as a different epitope. Antibodies were analyzed by SDS-PAGE, and the titer of mAbs was detected using an indirect ELISA.

### 2.4. Expression of MCP

The MCP gene of ISKNV was amplified and then ligated into the BamHI and EcoRI sites of the pET32a vector. Recombinant plasmids were then transformed into *Escherichia coli* BL21 cells as a kit (TRANS CD201, Beijing, China). Transformed cells were inoculated in LB broth containing AMP (100 µg/mL^−1^) and cultured at 37 °C by shaking at 200 rpm until the OD600 of the culture reached 0.6. Isopropyl-β-D-Thiogalactopyranoside (IPTG) was added to the culture, and the culture was incubated at 30 °C by shaking (200 rpm) for 3 h. Then, the expressed fusion protein was purified by affinity chromatography. Its concentration was measured by ultraviolet (UV) light absorption spectrophotometry, and it was stored at −20 °C.

### 2.5. Indirect Immunofluorescence Assay (IFA)

CPB cells were infected with ISKNV at an MOI of 1 in L-15 medium containing 5% FBS and were cultured at 28 °C for 48 h. Then the cells were fixed with 80% acetone for 30 min. After washing 3 times with PBS, the cells were incubated with monoclonal antibodies 3B12 6B3 (1:100) against ISKNV for 1 h. Then cells were washed 3 times with PBS and incubated with FITC-conjugated Rabbit anti-mouse IgG for 1 h. After the cells were washed 3 times with PBS, the cells were observed with the Leica DMI3000B fluorescence microscope.

### 2.6. Establishment of a Double-Antibody Sandwich ELISA

MCP was used to prepare the standard curve of the double-antibody sandwich ELISA. The capture mAbs were added into a 96-well microplate (100 µL/well) and were incubated overnight at 4 °C. Then the wells were blocked with 2% BSA at 37 °C for 2 h. Then purified MCP (two-fold serial dilutions) was added to the wells (100 µL/well) and incubated at 37 °C for 2 h. After washing 3 times with PBST, the different class of mAbs (Biotin-mAbs) used as the detection antibody was added to the wells used as detection antibodies were added to the wells (100 µL/well, diluted 1:100) and was incubated at 37 °C for 2 h. The wells were washed 3 times with PBST, and then horseradish peroxidase-labeled avidin (HRP-avidin, diluted 1:100) was added to the wells (100 µL/well) and was incubated at 37 °C for 1 h. The wells were then washed 5 times with PBST, and a TMB substrate solution was added to wells for 15 min. This reaction was stopped by the addition of 2 M H_2_SO_4_, and the absorbance was measured at 450 nm using an ELISA plate reader. The negative control (2% BSA), as well as the relationship between A450 nm and the MCP antigen concentration, was analyzed by Curve Expert 1.3. 

### 2.7. Validation of the Double-Antibody Sandwich ELISA

The viral titer was measured from three different batches of ISKNV. Then the virus was inactivated. The antigen concentration of the inactivated virus was determined using the established double-antibody sandwich ELISA. 

## 3. Results

### 3.1. Virus Purification

**Figure 1:**
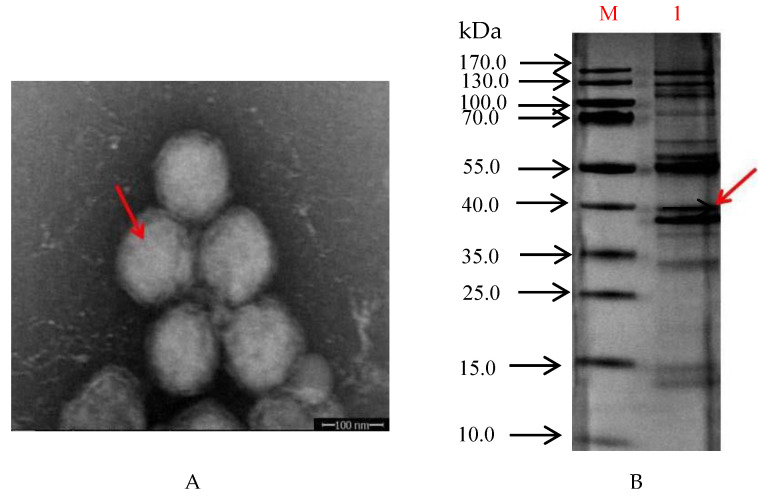
(**A**) Image of purified ISKNV by Electron microscopy negative staining (11,500×), Arrows refer to purified ISKNV. (**B**) Purified ISKNV SDS-PAGE; M: Protein Marker; 1: Purified ISKNV; Arrows refer to SDS-PAGE analysis of purified ISKNV.

The purified ISKNV particles primarily resided between the 50% and 60% sucrose gradients. Electron microscopy showed that the viral particles were mainly spherical, but some of the viral particles were nearly hexagonal (Figure 1A). The diameter of the viral particles was approximately 150 nm. SDS-PAGE analysis of purified ISKNV showed that the molecular weight of the purified ISKNV was distributed in the position of approximately 40 kDa to 55 kDa (Figure 1B and Figure S1).

### 3.2. Preparation of mAbs against ISKNV

#### 3.2.1. Generation of Hybridoma Cells

**Figure 2:**
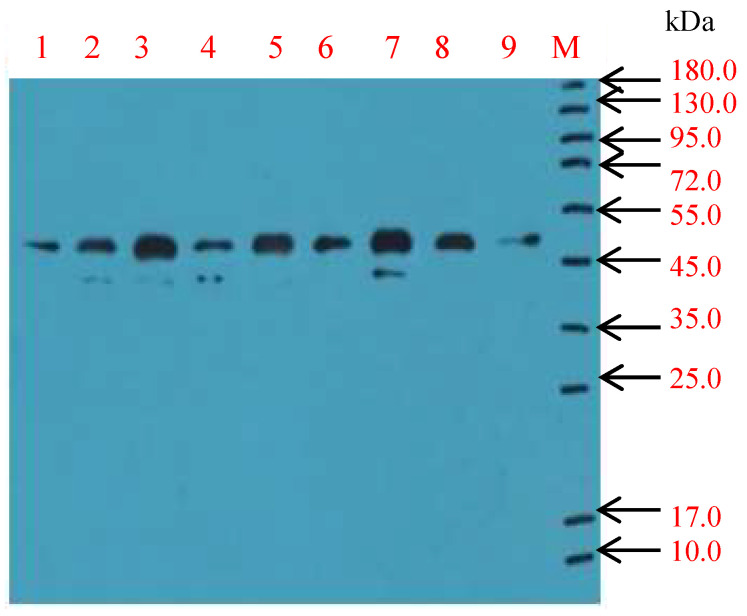
The supernatant of hybridoma cells was detected by Western blot. M: Protein, 1:2A4 5C2, 2: 2G5 5D5, 3: 3B12 6B3, 4: 8C9 3C12, 5: 1C8 1B9, 6: 8D4 6F5, 7: 9C9 2D7 and 8: 11F7 4C3; 9: Mouse antiserum.

Mice were intraperitoneally injected with purified ISKNV virus four times, and then the spleen cells of BALB/c mice were used for fusion. Through detection and screening, eight hybridoma cell strains stably secreting monoclonal antibodies against ISKNV were obtained and designated as 2A4 5C2, 2G5 5D5, 3B12 6B3, 8C9 3C12, 1C8 1B9, 8D4 6F5, 9C9 2D7 and 11F7 4C3, respectively (Figure 2 and Figure S2). Among them, 3B12 6B3 and 1C8 1B9 cell lines secreted higher titers of antibodies against ISKNV with titers of more than 1:1000. Additionally, there was no significant decrease in titers during propagation, indicating that the hybridomas cells had stable antibody secretion ability.

#### 3.2.2. Purification and SDS-PAGE of the mAbs

**Figure 3:**
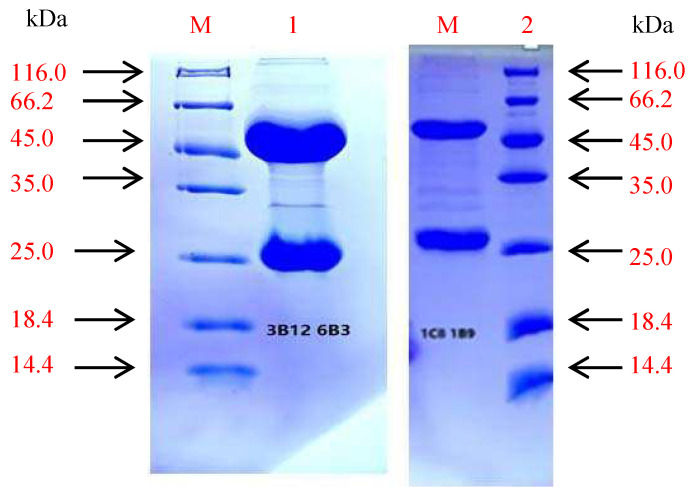
The purified monoclonal antibody was identified by SDS-PAGE. M: Protein Marker; 1: Purified monoclonal antibody: 3B12 6B3; 2: Purified monoclonal antibody: 1C8 1B9.

The mAbs were purified by affinity chromatography, and purified mAbs showed two main protein bands by SDS-PAGE analysis. One band was the heavy chain (approximately 45 kDa), and the other one was the light chain (approximately 25 kDa) (Figure 3 and Figure S3). Monoclonal antibodies from 1C8 1B9 and 3B12 6B3 were purified, and the protein concentration was 7.5 mg/mL and 2.1 mg/mL, respectively.

#### 3.2.3. Characterization of the mAbs

There were different subtypes of IgG. The subclass of the mAbs was determined using the mouse monoclonal antibody isotyping kit (Sigma), and the results showed that antibodies 1C8 1B9 and 8D4 6F5 belonged to the IgG2b class, and the others belonged to the IgG1 class. The 3B12 6B3 antibody was labeled with biotin and used as a detection antibody to analyze the epitopes of antibodies in the cell supernatant. As described in the kit, when the OD value was more than 1.5, it was regarded as a different epitope compared with 3B126B3. The test results showed that 8C9 3C12, 11F7 4C3 and 3B12 6B3 were the same epitopes, and the others belonged to different epitopes (Table 2 and Table 3).

### 3.3. Expression and Purification of MCP

**Figure 4:**
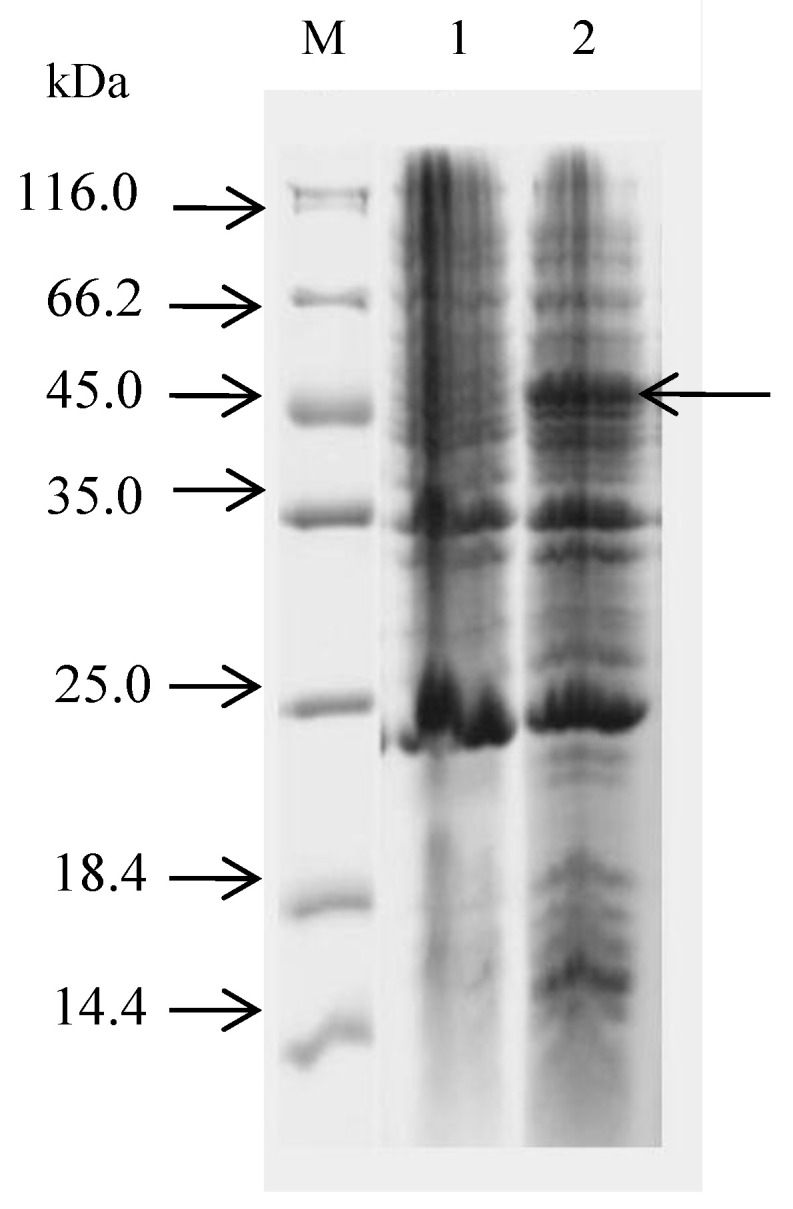
Electrophoresis of whole strains of positive strains. M: Protein Marker; 1: non-IPTG-induced whole cell protein, 2: IPTG-induced whole cell protein; Arrows refer to 45 kDa recombinant proteins.

The MCP gene was amplified and ligated into the pET32a vector, which was transformed into *E. coli* BL21 cells. The expressed fusion protein was analyzed by SDS-PAGE. The result showed that the molecular weight of MCP was approximately 45 kDa (Figure 4 and Figure S4).

### 3.4. Indirect Immunofluorescence Assay (IFA)

**Figure 5:**
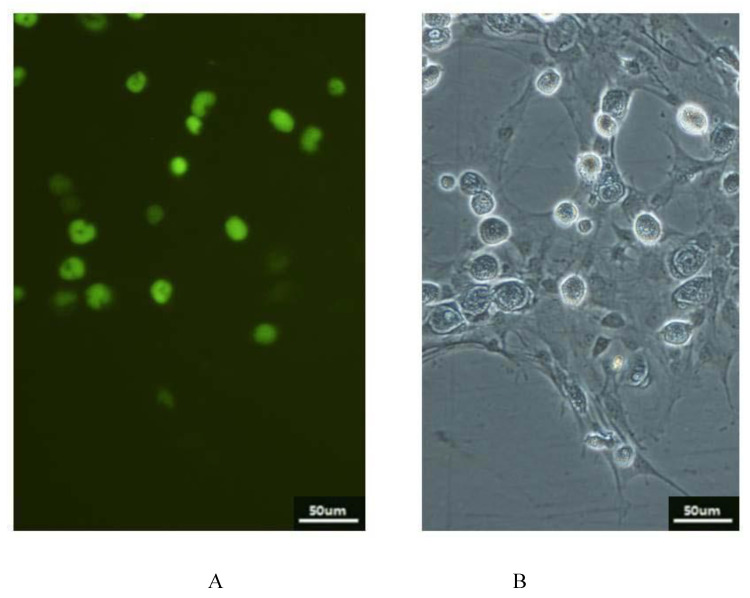
Indirect immunofluorescence assay (IFA). (**A**) The cells incubated with monoclonal antibody and observed under the fluorescence microscope; (**B**) The cells observed under the phase-contrast microscope.

The ISKNV-infected CPB cells were incubated with monoclonal antibodies and FITC-conjugated rabbit anti-mouse IgG, and then were observed with the fluorescence microscope (Figure 5).

### 3.5. Establishment of a Double-Antibody Sandwich ELISA

**Figure 6:**
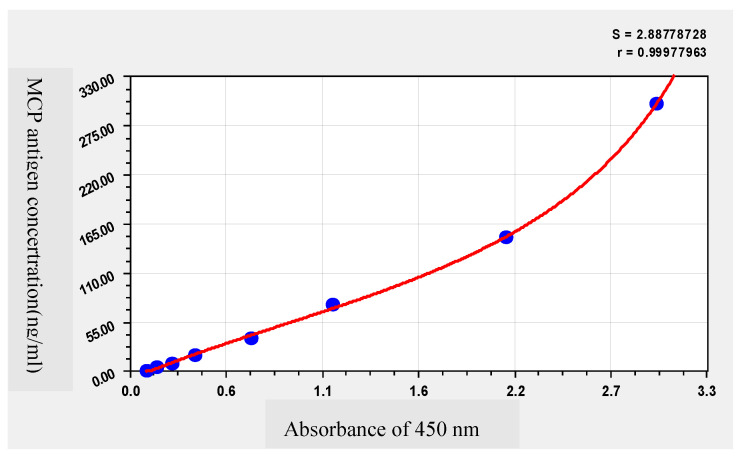
Standard curve of effective antigen quantification of inactivated ISKNV. Detection of MCP protein with concentrations of 4.69, 9.38, 18.75, 37.5, 75, 150 and 300 ng/mL.

A double-antibody sandwich ELISA was established using the mAb 1C8 1B9 (1 ug/mL) as the capture antibody and Biotin-labeled 3B12 6B3 as the detection antibody. The linear standard curve was obtained between the value of A450nm and the MCP antigen concentration, and the linear equation was y = 17.6480x − 2.1091, R^2^ = 0.99 (Figure 6). The linear range of this method was 4.69~300 ng/mL and the sensitivity were up to 0.9 ng/mL.

### 3.6. Validation of the Double-Antibody Sandwich ELISA in Inactivated ISKNV Vaccine 

The MCP antigen concentration of three batches of inactivated ISKNV vaccines was detected using the double-antibody sandwich ELISA. The antigen concentration (263.79, 287.27, 111.63 ng/mL) was in parallel with the ISKNV titer before inactivation (10^−8.5^, 10^−9.25^, 10^−8.14^ TCID _50_) (Table 4). The results showed that the MCP antigen concentration of inactivated ISKNV vaccines was positively correlated with TCID _50_ of ISKNV, and this double-antibody sandwich ELISA might be used to evaluate the quality of inactivated ISKNV vaccines.

## 4. Discussion

ISKNV causes severe systemic diseases with high morbidity and mortality in Chinese perch. Vaccination is the most effective method used to prevent and control these diseases [8]. The quality evaluation of inactivated whole-virus vaccines is very important for developing an efficient vaccine. In this study, mouse monoclonal antibodies against ISKNV were generated and selected as the coating antibody and the capture antibody, respectively. Then we developed an antibody sandwich ELISA assay to detect the MCP antigen concentration in the inactivated ISKNV vaccine. The results showed that this assay was sensitive, convenient and accurate in the detection of antigen concentrations in inactivated ISKNV vaccines. This assay provided a useful tool for the detection of antigen quality for ISKNV inactivated vaccines. 

The current primary method for testing the potency of vaccines is the challenge test, which is costly and labor-intensive. The enzyme-linked immunosorbent assay (ELISA) is a specific, sensitive and convenient method for measuring macromolecular proteins [9]. 

In this study, we established a double-antibody sandwich ELISA for the detection of antigen quality in the killed or inactivated ISKNV vaccine. The linear range of this method was 4.69~300 ng/mL, and the sensitivity was up to 0.9 ng/mL. Compared to a polyclonal antibody, MAb-based assays are stable and specific for detecting because Mabs from stable hybridoma clones ensured a continuous supply of large quantities of well-characterized antibodies against unique epitopes [10]. In the study, two monoclonal antibodies (1C8 1B9 and 3B12 6B3) against ISKNV were obtained and used as the capture antibody/detection antibody, respectively. It is more sensitive when different class antibodies were used as the detection and capture antibodies with a broad spectrum of epitopes. Furthermore, the detection of multiple epitopes offers better sensitivity for detecting proteins that are present in low concentrations in a sample [11,12]. The DAS ELISA was established to determine the effective antigen yields in inactivated vaccines and thus represents an alternative for assessing the potency of FMD vaccines in vitro [13,14]. A great advantage of the ELISA method is that it can detect viruses in very low concentrations (1–10 ug/mL), with only a few antibodies needed [15]. The ELISA was sensitive. Additionally, it was also used to detect Strongyloides immune complexes in a group of elderly people, and it detected six times (27.27%) more positive samples compared to conventional parasitological techniques (4.54%) [16]. Moreover, the double-antibody sandwich ELISA is more sensitive, convenient and accurate in the detection of antigen quality. The major capsid protein (MCP) is the most abundant protein in ISKNV and is the primary protective antigen protein. The major capsid protein (MCP) (ORF006) is an important structural component that mediates virus entry into the host cell; therefore, it is a good candidate antigen of ISKNV for subunit vaccine development [17]. The major capsid protein (MCP) is the predominant structural component of the iridovirus particles, comprising 40–45% of the total particle poly-peptide, and MCP seems to be highly conserved [18,19]. Thus, MCP was expressed and purified as the standard protein sample, and it showed good linearity in the detection of the antigen quality. 

In the sample tests, three batches of inactivated vaccines were detected using the double-antibody sandwich ELISA, and viral titers were measured before inactivation. The results showed that there was a positive correlation between the antigen contents and the viral titers. The antigen content can sensitively show the change of virus titer, which shows that this method is reliable. Additionally, the antigen-capture double-antibody sandwich ELISA can be used to quantitatively determine the concentration of MCP in vaccines. Thus, this double-antibody sandwich ELISA provides a highly sensitive and specific method for determining the antigen quality of the inactivated ISKNV vaccine.

## 5. Conclusions

In conclusion, we developed a highly sensitive and specific antigen-capture double-antibody sandwich ELISA for determining MCP antigen concentration in inactivated ISKNV using two different classes of antibodies. The double-antibody sandwich ELISA provides a useful tool for the quantitative detection of antigens in the inactivated ISKNV vaccine.

## Figures and Tables

**Table 1:** Animal immunization.

Immunization Times	Immunogen Preparation	Immunization Route	Immune Cycle	Immunizing Dose
First immunization	immunogen + Freund’s complete adjuvant	intraperitoneally injected	2-week intervals	50–100 μg/mice
Second immunization	immunogen + Freund’s incomplete adjuvant	intraperitoneally injected	2-week intervals	50–80 μg/mice
Third immunization	immunogen + Freund’s incomplete adjuvant	intraperitoneally injected	2-week intervals	50–80 μg/mice
fourth immunization	immunogen + Freund’s incomplete adjuvant	intraperitoneally injected	1-week intervals	50–80 μg/mice

**Table 2:** Class-subclass-type assay of McAbs.

Cell Lines	1C8 1B9	2A4 5C2	2G5 5D5	3B1 26B3	8C9 3C12	8D4 6F5	9C9 2D7	11F7 4C3
Subclasses	IgG2b	IgG1	IgG1	IgG1	IgG1	IgG2b	IgG1	IgG1

**Table 3:** Epitope identification.

Cell Lines	1C8 1B9	2A4 5C2	2G5 5D5	8C9 3C12	9C9 2D7	11F7 4C3
OD	1.9874	1.8386	1.9723	0.2078	2.0015	0.1068

**Table 4:** Detection results of antigen quantification of different batches of ISKNV vaccine (ng/mL).

Batches of Vaccine	OD Mean	Sample Concerntration (ng/mL)	TCID _50_
A	3.68	263.79	10^−8.5^
B	3.82	287.27	10^−9.25^
C	2.70	111.63	10^−8.14^

## Data Availability

The study details can be found in the paper.

## Supplementary Materials

The following are available online at https://www.mdpi.com/article/10.3390/vaccines9111264/s1, Figure S1: Uncropped blot: Purified ISKNV SDS-PAGE, Figure S2: Uncropped blot: The supernatant of hybridoma cells was detected by Western blot, Figure S3: Uncropped blot: The purified monoclonal antibody was identified by SDS-PAGE, Figure S4: Uncropped blot: Electrophoresis of whole strains of positive strains.
