# Supplementary material for: Development of a Double-Antibody Sandwich ELISA for Rapid Detection of the MCP Antigen Concentration in Inactivated ISKNV Vaccines
Source: Vaccines (Basel). 2021 Nov 2;9(11):1264. doi: 10.3390/vaccines9111264 (PMC8623861; doi:10.3390/vaccines9111264)
Supplement: Supplementary file 1 [file vaccines-09-01264-s001.zip › vaccines-1355723 supplementary.pdf]

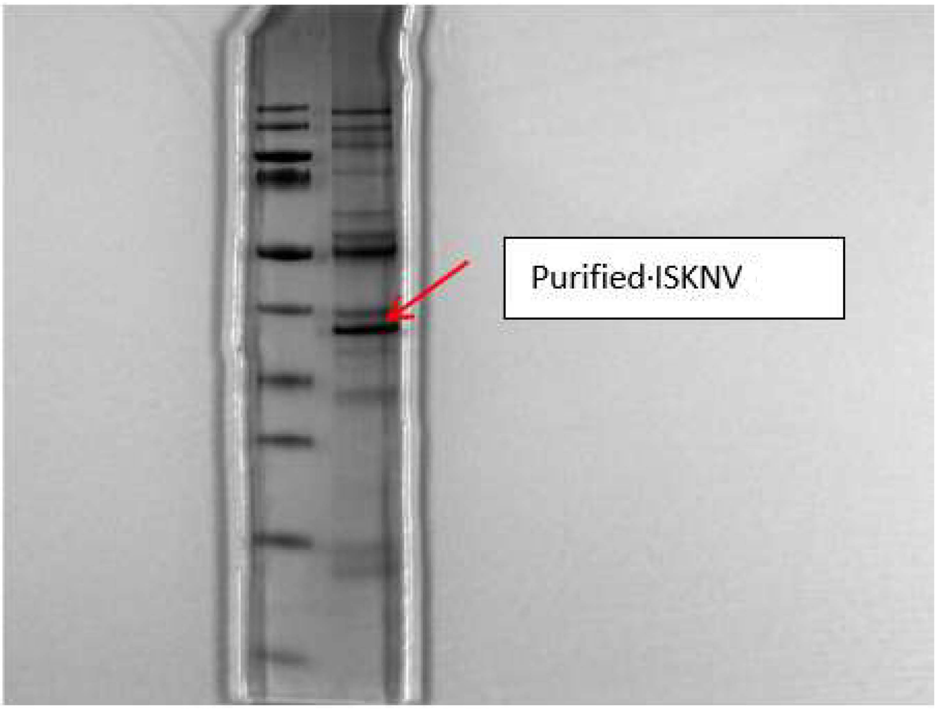

Figure S1. Uncropped blot: Purified ISKNV SDS-PAGE.

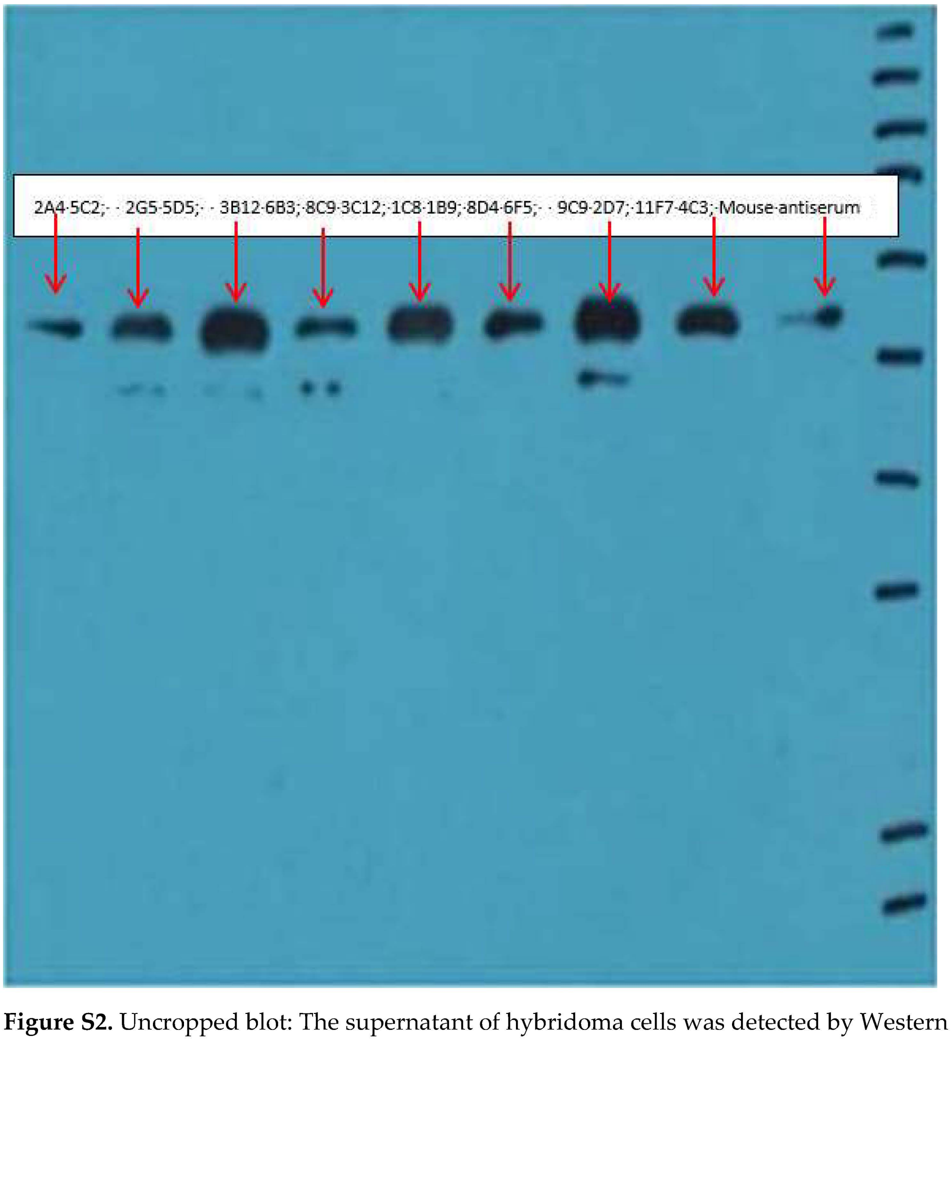

Figure S2. Uncropped blot: The supernatant of hybridoma cells was detected by Western blot.

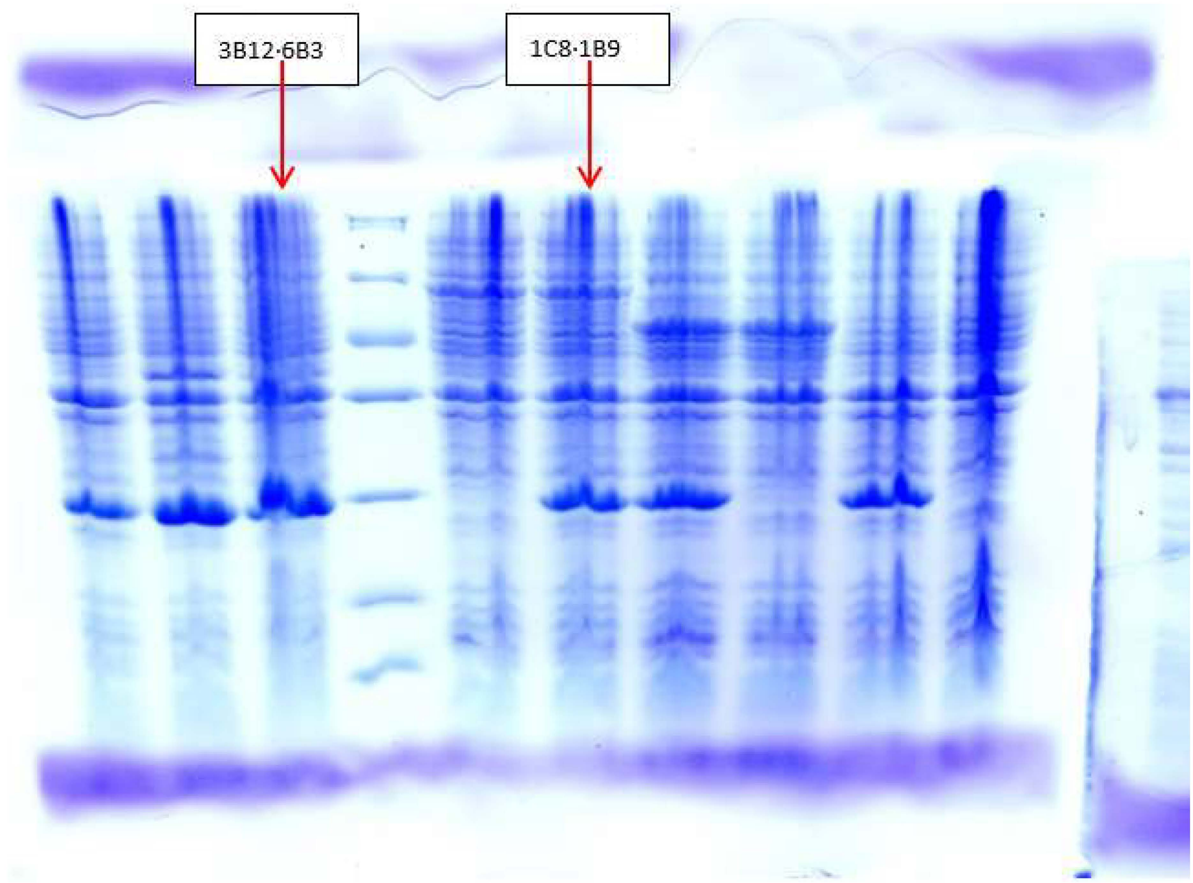

Figure S3. Uncropped blot: The purified monoclonal antibody was identified by SDS-PAGE.

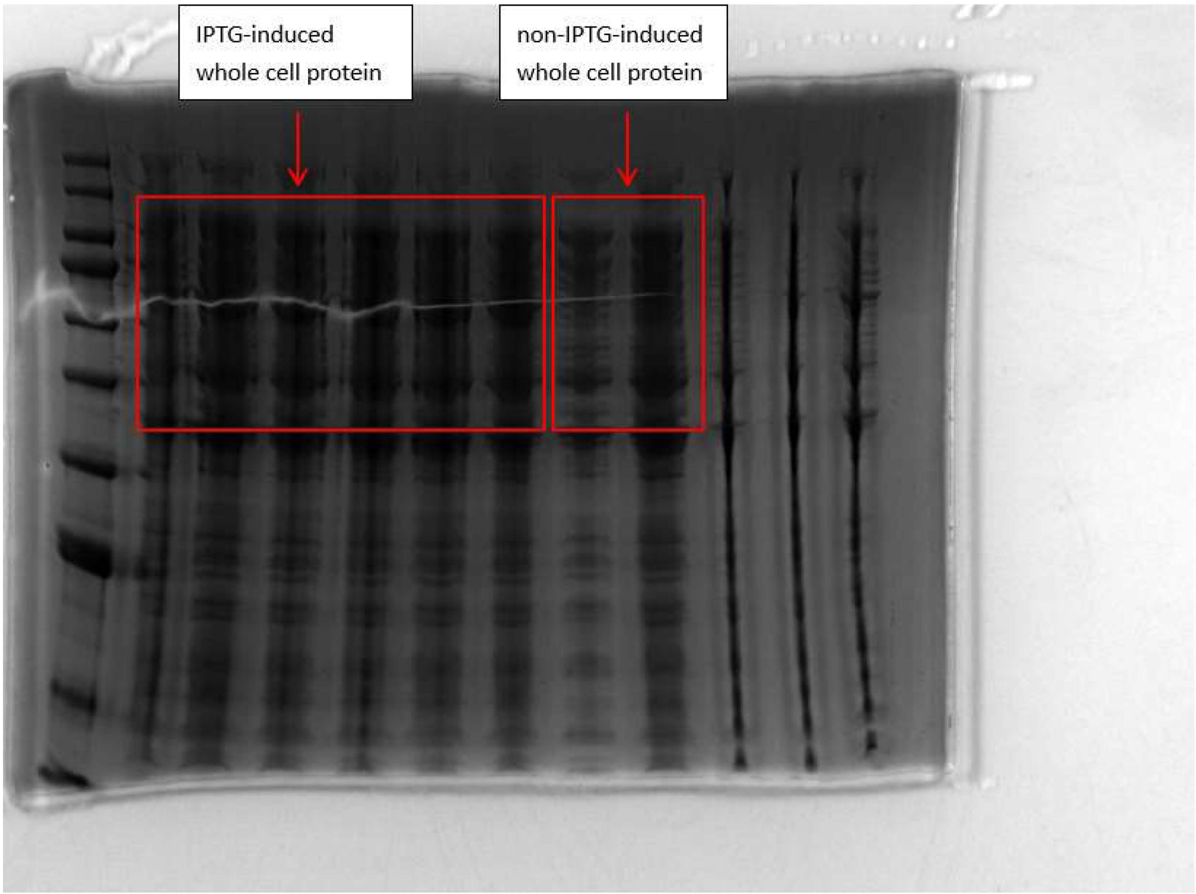

Figure S4. Uncropped blot: Electrophoresis of whole strains of positive strains.
